# The Use of Step-Down Oral Antibiotic Therapy for Uncomplicated Gram-Negative Bacteremia and Risk Factors for Adverse Outcomes

**DOI:** 10.1093/ofid/ofag200

**Published:** 2026-04-09

**Authors:** Ruihong Luo, Elijah D Wade, Ruth Ann Bertsch, Dana S Clutter, Jacek Skarbinski

**Affiliations:** Division of Infectious Diseases, Kaiser Permanente Sacramento Medical Center, Sacramento, California, USA; Division of Research, Kaiser Permanente Northern California, Pleasanton, California, USA; Division of Hospital Based Services, Kaiser Permanente Sacramento Medical Center, Sacramento, California, USA; Division of Infectious Diseases, Kaiser Permanente South SanFrancisco Medical Center, South San Francisco, California, USA; Division of Infectious Diseases, Kaiser Permanente Oakland Medical Center, Oakland, California, USA; Division of Research, Kaiser Permanente Northern California, Pleasanton, California, USA

**Keywords:** administration route, antibiotic treatment, gram-negative bacteremia, risk factors, step-down oral therapy

## Abstract

**Background:**

Step-down oral antibiotic therapy (SDO) is commonly used to treat uncomplicated gram-negative bacteremia (GNB), but real-world treatment outcomes and risk factors for failure remain unclear.

**Methods:**

We conducted a retrospective cohort study of hospitalized adults with uncomplicated GNB within a large integrated healthcare system from 2018 to 2022, stratifying patients by antibiotic route: complete course of intravenous antibiotic (CIV) or initial intravenous followed by SDO. The primary outcome was a composite of 90-day all-cause mortality, all-cause readmission, or recurrent GNB. Secondary analysis identified risk factors for adverse outcomes using multivariable Cox regression.

**Results:**

We included 9164 patients, of whom 8263 received SDO and 901 received CIV therapy. All-cause mortality was 1.1% overall, with no significant difference between groups (CIV 1.4% vs SDO 1.0%, *P* = .3). All-cause readmission and recurrent GNB were more frequent in the CIV group than SDO group (33% vs 18%, and 5.8% vs 2.4%, respectively; both *P* < .001). A high Charlson Comorbidity Index (CCI) was independently associated with all adverse outcomes in the full cohort and in both treatment groups. Among SDO patients, fluoroquinolones were associated with a lower GNB recurrence rate (1.6%), whereas beta-lactams were linked to a higher recurrence rate (3.2%).

**Conclusions:**

Step-down oral antibiotic therapy appears as effective as CIV for treating uncomplicated GNB, with comparable mortality. A high CCI score is a strong predictor of adverse outcomes. Fluoroquinolones remain a favorable SDO option, though further research is warranted to evaluate alternative oral antibiotic agents.

Gram-negative bacteremia (GNB) is a major cause of morbidity and mortality in hospitals, most often originating from the urinary and gastrointestinal tracts [1], with *Escherichia coli* and *Klebsiella pneumoniae* as the most frequently identified pathogens [2]. Treatment has traditionally involved extended IV antibiotic courses, often lasting 2 weeks or more, depending on complications [3]. Recent evidence supports transitioning to oral antibiotics following source control and clinical stability, with studies demonstrating comparable outcomes to intravenous (IV)-only therapy, along with reduced hospital stays and costs [2–6]. Despite these findings, standardized treatment guidelines remain absent. At Kaiser Permanente Northern California (KPNC), step-down oral (SDO) antibiotic therapy is often used for patients with uncomplicated GNB. This study compares outcomes between patients treated exclusively with IV antibiotics and those who transitioned to oral therapy, while evaluating risk factors for adverse outcomes based on patient comorbidities, the type of gram-negative pathogen, and antibiotic regimen employed.

## METHODS

### Study Setting and Patient Selection

We conducted a retrospective cohort study of adults (≥18 years) hospitalized with GNB at KPNC from 1 January 2018, to 31 December 2022. All included patients had a positive blood culture for gram-negative bacteria during the index hospitalization, and no documented GNB in the prior year. Continuous KPNC membership for ≥1 year before and ≥90 days after the index hospitalization was required to ensure adequate characterization within the database.

Exclusion criteria included the following: (1) blood cultures isolating non–gram-negative organisms in addition to gram-negative bacteria, as polymicrobial bacteremia may require different management and is associated with outcomes distinct from uncomplicated GNB; (2) death during the index hospitalization, as these cases were more likely to represent complicated infections and should not be classified as uncomplicated GNB; (3) absence of intravenous antibiotic therapy during hospitalization, since the study aimed to evaluate step-down from IV to oral antibiotic therapy in patients initially treated with IV agents; (4) presence of complications such as deep abscesses (including brain, lung, liver, spleen, intra-abdominal, pelvic, or paraspinal abscesses), endocarditis, discitis, osteomyelitis, septic arthritis, or prosthetic or surgical site infections, which are considered complicated GNB; (5) total antibiotic duration ≥21 days or hospitalization ≥21 days, as prolonged antibiotic treatment or hospitalization may indicate clinically suspected complicated GNB; and (6) discharge to hospice or skilled nursing facilities, as follow-up data for these patients were incomplete in the KPNC database.

### Data Extraction

Microbiologic data, along with patient demographic information, clinical characteristics, and outcomes, were extracted from the KPNC electronic health record (EHR). A text string–based search of blood culture reports within the EHR was used to identify gram-negative organisms from positive blood culture results. Antibiotic data were obtained from KPNC pharmacy databases.

Patients were stratified into 2 groups based on the route of antibiotic administration: complete course of intravenous antibiotics (CIV) and step-down oral (SDO). The CIV group received IV antibiotics exclusively for the treatment of GNB, whereas the SDO group initiated therapy with IV antibiotics and subsequently transitioned to oral antibiotics to complete treatment. Upon hospital discharge, patients in the CIV group continued IV antibiotic therapy through the Outpatient Parenteral Antimicrobial Therapy (OPAT) program, whereas patients in the SDO group received all prescribed oral medications from the discharge pharmacy, with instructions provided by a pharmacist.

IV and oral antibiotics were categorized by agent and clinical indication according to the institutional antibiotic stewardship protocol of KPNC (Table 1).

**Table 1.** Antibiotic Categories

Antibiotic Route	Categories	Antibiotic Agent	Related Clinical Significance
Intravenous	IV-Group 1	Cefazolin, ceftriaxone, ceftaroline, ampicillin-sulbactam	Agents active against pathogens that are susceptible to beta-lactams.
	IV-Group 2	Cefepime, ceftazidime, piperacillin-tazobactam	Agents active against *Pseudomonas species* and pathogens resistant to Group 1 agents.
	IV-Group 3	Ertapenem, meropenem, gentamicin, amikacin, ceftazidime-avibactam, ceftolozane-tazobactam, meropenem-vaborbactam, cefiderocol	Agents active against multidrug-resistant pathogens that are resistant to both Group 1 and Group 2 agents.
	IV-Group 4	Fluoroquinolones, including ciprofloxacin, levofloxacin, moxifloxacin	Agents active against pathogens resistant to beta-lactams but susceptible to fluoroquinolones, and/or agents used in patients with beta-lactam allergies.
	IV-Group 5	Aztreonam	Used for patients with allergies to other classes of antibiotics
	IV-Group 6	Multiple IV antibiotics	Received multiple IV antibiotics concurrently
Oral	PO-Group 1	Ciprofloxacin, levofloxacin, moxifloxacin	Oral fluoroquinolones
	PO-Group 2	Cephalexin, cefadroxil, cefdinir, cefpodoxime, amoxicillin, amoxicillin-clavulanic	Oral beta-lactams
	PO-Group 3	Trimethoprim-sulfamethoxazole (TMP-SMX)	**…**
	PO-Group 4	Others	Agents not in PO-Group 1, PO-Group 2 or PO-Group 3
	PO-Group 5	Multiple oral antibiotics	Received multiple oral antibiotics concurrently

### Outcomes

All patients were followed for 90 days after discharge from the index hospitalization. The primary outcome comprised 3 components: all-cause mortality, all-cause readmission, and recurrent GNB, which was defined as a positive blood culture for the same pathogen during the follow-up period. Each outcome was evaluated individually, both in the overall cohort and within subgroups. Risk factors associated with each individual outcome were analyzed separately.

### Statistical Analysis

The demographics (age, sex, race) and clinical characteristics (length of hospital stay [LOS], intensive care unit [ICU] admission, Charlson Comorbidity Index [CCI] [7], Laboratory Acute Physiology [LAP] Score, antibiotic category, and GNB pathogen) were summarized for the full cohort and stratified by treatment group (CIV vs SDO). Categorical variables were presented as counts and percentages, while continuous variables were reported as medians with interquartile ranges. Group differences were assessed using the Chi-square or Fisher's exact test for categorical variables, and the Student's t-tests or Kruskal–Wallis test for continuous variables, as appropriate.

Cox proportional hazards regression was used to evaluate the association between antibiotic route (CIV vs SDO) and the 3 components of primary outcome. Variables with a *P*-value < .05 in bivariate analyses were included in the multivariable model, and only those that remained statistically significant (*P* < .05) were retained in the final model. All models were adjusted for age, sex, CCI, and the presence of cancer. Additional bivariate analyses were performed to assess the impact of the causative GNB pathogen and the antibiotic class on outcomes within each treatment group. Subgroup analyses were conducted to compare characteristics of patients with differing outcomes (eg, death vs survival, readmission vs no readmission, recurrent GNB vs no recurrence). Separate Cox proportional hazards regression models were also developed to identify risk factors for adverse outcomes within the CIV and SDO groups.

## RESULTS

### Demography

**Figure 1.**
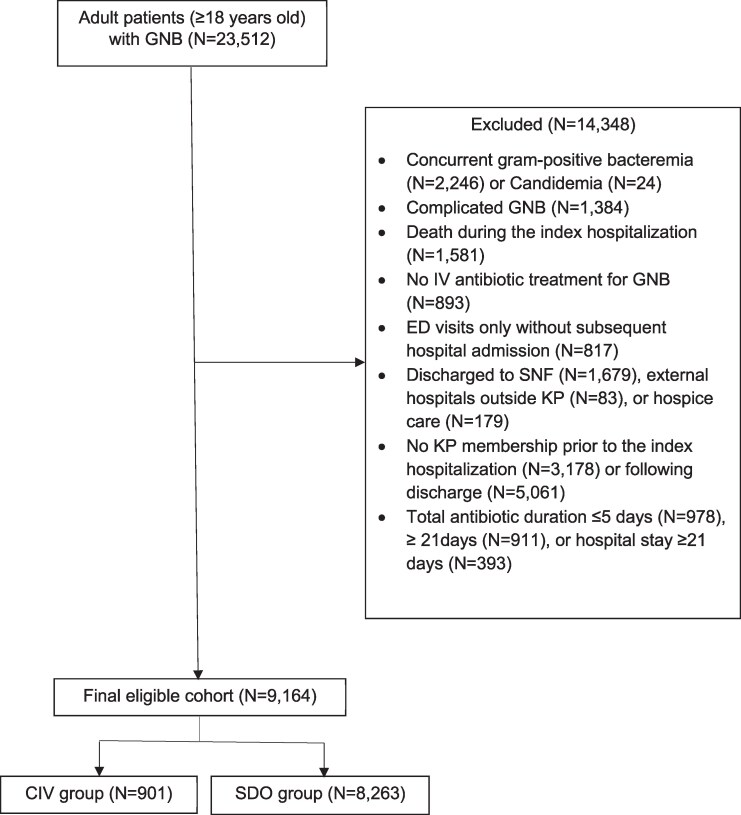
CONSORT flow diagram illustrating patient exclusion and group assignment. Abbreviations: CIV, complete course of intravenous antibiotics; ED, emergent department; GNB, gram-negative bacteremia; IV, intravenous; KP, Kaiser Permanente; SDO, step-down oral; SNF, skilled nursing facility.

During the study period, 23 512 patients with GNB were identified. After applying exclusion criteria, 9164 patients were included in the final analysis, comprising 8263 in the SDO group and 901 in the CIV group (Figure 1).

In the full cohort, the median age was 72 years (IQR: 61–81), with 58% female and 42% male. The median LOS was 5 days (IQR: 4–6), and 16.1% (n = 1477) of patients required ICU admission, with a median ICU stay of 3 days (IQR: 2–4). Based on the CCI, 52% (n = 2762) had mild comorbidity (CCI 0–2), 19% (n = 1761) had moderate comorbidity (CCI 3–4), and 29% (n = 2641) had severe comorbidity (CCI ≥5). *Escherichia coli* was the most frequently isolated pathogen, accounting for 68% (n = 6197) of GNB cases, followed by *Klebsiella species (spp.)* (13%, n = 1180), *Proteus spp.* (3.9%, n = 335), *Pseudomonas spp.* (3.4%, n = 309), and *Enterobacter spp.* (2.2%, n = 206). Polymicrobial GNB was identified in 2.1% (n = 194) of cases.

Patients in the SDO group were generally 2 years older than those in the CIV group, with a higher proportion of females and White individuals. They also had lower CCI and LAP scores. *Escherichia coli*, *Proteus spp.*, and *Klebsiella spp.* were more frequently isolated in the SDO group, whereas *Enterobacter spp.*, *Pseudomonas spp.*, and *Serratia spp.* were more prevalent in the CIV group (Table 2).

**Table 2.** Comparison of Demographic and Clinical Characteristics Between CIV and SDO Groups

Characteristics	CIV Group N = 901^a^	SDO Group N = 8,263^a^	*P* Value^b^	Overall N = 9,164^a^
Index age	70 (61, 80)	72 (60, 81)	.015	72 (61, 81)
Sex			<.001	
Female	462 (51%)	4891 (59%)		5353 (58%)
Male	439 (49%)	3372 (41%)		3811 (42%)
Race/ethnicity			.32	
White	438 (49%)	4307 (52%)		4745 (52%)
Asian	172 (19%)	1367 (17%)		1539 (17%)
Black	65 (7.2%)	572 (6.9%)		637 (7.0%)
Hispanic	204 (23%)	1827 (22%)		2031 (22%)
Other/multiracial	21 (2.3%)	182 (2.2%)		203 (2.2%)
Unknown	1 (0.1%)	8 (<0.1%)		9 (<0.1%)
Length of hospital stay (d)	7.00 (5.00, 10.00)	5.00 (4.00, 6.00)	<.001	5.00 (4.00, 6.00)
Length of inpatient IV antibiotics (d)	6.00 (5.00, 8.00)	4.00 (3.00, 5.00)	<.001	4.00 (3.00, 6.00)
Total antibiotic duration (d)	14.0 (10.0, 16.0)	14.0 (11.0, 15.0)	<.001	14.0 (11.0, 15.0)
ICU admission	240 (26.6%)	1237 (15%)	<.001	1477 (16.1%)
Length of ICU stay (d)	3.00 (2.00, 4.00)	2.00 (2.00, 3.00)	<.001	3.00 (2.00, 4.00)
Charlson Comorbidity Index Score			<.001	
Mild (1–2)	369 (41%)	4393 (53%)		4762 (52%)
Moderate (3–4)	184 (20%)	1577 (19%)		1761 (19%)
Severe (≥5)	348 (39%)	2293 (28%)		2641 (29%)
Laboratory acute physiology score	96 (71, 122)	90 (67, 114)	<.001	90 (67 115)
Pathogen of GNB			<.001	
*Escherichia coli*	557 (62%)	5640 (68%)		6197 (68%)
*Citrobacter spp.*	13 (1.4%)	105 (1.3%)		118 (1.3%)
*Enterobacter spp.*	25 (2.8%)	181 (2.2%)		206 (2.2%)
*Haemophilus spp.*	10 (1.1%)	78 (0.9%)		88 (1.0%)
*Klebsiella spp.*	86 (9.5%)	1094 (13%)		1180 (13%)
*Proteus spp.*	30 (3.3%)	325 (3.9%)		335 (3.9%)
*Pseudomonas spp.*	66 (7.3%)	243 (2.9%)		309 (3.4%)
*Serratia spp.*	19 (2.1%)	86 (1.0%)		105 (1.1%)
Multiple organisms	22 (2.4%)	172 (2.1%)		194 (2.1%)
Other	73 (8.1%)	339 (4.1%)		412 (4.5%)
Comorbidity of cancer			<.001	
No	617 (68%)	6172 (75%)		6789 (74%)
Yes	284 (32%)	2091 (25%)		2375 (26%)
Category of IV antibiotic				
IV-Group 1	125 (14%)	3472 (42%)		3597 (39%)
IV-Group 2	35 (3.9%)	561 (6.8%)		596 (6.5%)
IV-Group 3	39 (4.3%)	26 (0.3%)		65 (0.7%)
IV-Group 4	2 (0.2%)	230 (2.8%)		232 (2.5%)
IV-Group 5	2 (0.2%)	13 (0.2%)		15 (0.2%)
IV-Group 6	698 (77%)	3961 (48%)		4659 (51%)
Category of oral antibiotic				
PO-Group 1	…	3866 (47%)		3866 (42%)
PO-Group 2	…	3535 (43%)		3535 (39%)
PO-Group 3	…	269 (3.3%)		269 (2.9%)
PO-Group 4	…	2 (<0.1%)		2 (<0.1%)
PO-Group 5	…	591 (7.2%)		591 (6.4%)
None	901 (100%)	…		901 (9.8%)

^a^Median (IQR); N (%).

^b^Wilcoxon rank sum test; Fisher's Exact Test with simulated *P* value.

CIV, complete course of intravenous antibiotics; ICU, intensive care unit; SDO, step-down oral.

Hospitalization patterns differed notably between the 2 groups. Patients in the SDO group had shorter LOS and were less likely to require ICU admission. Among those admitted to the ICU, SDO patients also had shorter ICU stays compared to those in the CIV group (Table 2).

Both groups had a similar median total duration of antibiotic therapy (14 days; IQR 11–15); however, as expected, the SDO group received a shorter course of IV treatment. The most commonly used IV agents in both groups were the beta-lactams for pathogens without resistance (IV-Group 1). Among patients in the SDO group, 47% transitioned to fluoroquinolones (PO-Group 1), 43% to beta-lactams (PO-Group 2), and 3.3% to trimethoprim-sulfamethoxazole (TMP-SMX) (PO-Group 3) (Table 2).

### Treatment Outcomes by Antibiotic Route

In the unadjusted analysis during 90-day follow-up period, the all-cause mortality rate in the full cohort was 1.1% (98 deaths among 9164 patients), with no significant difference between the CIV group (1.4%; 13 deaths among 901 patients) and SDO groups (1.0%; 85 deaths among 8263 patients) (*P* = .3). All-cause readmission occurred in 19.2% of patients (1756 out of 9164) and was significantly more frequent in the CIV group (33.1%, 298 out of 901 patients) than in the SDO group (17.6%, 1458 out of 8263 patients) (*P* < .001). Recurrent GNB was observed in 2.7% of patients (248 out of 9164) and was also significantly higher in the CIV group (5.8%, 52 out of 901 patients) compared with SDO group (2.4%, 196 out of 8263 patients) (*P* < .001) (Table 3).

**Table 3.** Comparison of the Treatment Outcomes Between CIV and SDO Groups Within 90-Day Follow-Up

Outcomes	CIV Group N = 901	SDO Group N = 8263	*P* Value	Full Cohort N = 9164
All-cause mortality	13 (1.4%)	85 (1.0%)	.3	98 (1.1%)
All-cause readmission	298 (33.1%)	1458 (17.6%)	<.001	1756 (19.2%)
Recurrent GNB	52 (5.8%)	196 (2.4%)	<.001	248 (2.7%)

CIV, complete course of intravenous antibiotics; GNB, Gram-negative bacteremia; SDO, step-down oral.

Cox proportional hazards analysis showed no significant difference in mortality between CIV and SDO groups (HR = 1.24; 95% CI, .65–2.35; *P* = .5). However, the SDO group had significantly lower risks of both all-cause readmission (HR = 0.68; 95% CI, .59–.78; *P* < .001) and recurrent GNB (HR = 0.47; 95% CI, .33–.66; *P* < .001) compared to the CIV group (Table 4).

**Table 4.** Risk Factors for Various Outcomes Within 90-Day Follow-Up: Adjusted Cox Regression Analysis for Full Cohort

Characteristic	All-Cause Mortality		All-Cause Readmission		Recurrent GNB	
	HR (95% CI)	*P* Value	HR (95% CI)	*P* Value	HR (95% CI)	*P* Value
SDO	1.24 (.65, 2.35)	.5	0.68 (.59, .78)	<.001	0.47 (.33, .66)	<.001
Index age	1.00 (.99, 1.02)	.8	1.00 (.99, 1.00)	.2	0.99 (.98, 1.00)	.095
Sex						
Female	…	…	…	…	…	…
Male	1.78 (1.18, 2.69)	.006	1.28 (1.16, 1.41)	<.001	1.60 (1.24, 2.06)	<.001
Race/ethnicity						
White	…	…	…	…	…	…
Asian	1.35 (.78, 2.31)	.3	0.92 (.71, 1.19)	.5	1.24 (.88, 1.75)	.2
Black	0.58 (.21, 1.61)	.3	1.08 (.68, 1.71)	.7	1.24 (.78, 1.99)	.4
Hispanic	0.91 (.52, 1.60)	.7	0.93 (.29, 2.97)	>.9	1.15 (.83, 1.58)	.4
Other/multiracial	2.02 (.62, 6.57)	.2	0.35 (.00, 2,838,461)	.9	0.61 (.19, 1.91)	.4
Charlson Comorbidity Index						
Mild (0–2)	…	…	…	…	…	…
Moderate (3–4)	2.80 (1.44, 5.43)	.002	1.53 (1.34, 1.75)	<.001	1.60 (1.12, 2.29)	.011
Severe (≥ 5)	4.15 (2.30, 7.50)	<.001	2.02 (1.79, 2.27)	<.001	2.25 (1.64, 3.08)	<.001
LOS	1.01 (.85, 1.19)	>.9	1.06 (1.03, 1.10)	.002	1.05 (.95, 1.16)	.4
Inpatient IV antibiotic duration	1.09 (.89, 1.32)	.4	1.00 (.96, 1.05)	.9	0.98 (.86, 1.10)	.7
ICU admission	1.41 (.86, 2.32)	.2	0.97 (.85, 1.10)	.7	1.10 (.79, 1.54)	.6
Cancer	2.68 (1.74, 4.13)	<.001	1.26 (1.13, 1.40)	<.001	1.05 (.95, 1.52)	.3

HR, hazard ratio; LOS, length of hospital stay; SDO, step-down oral antibiotic therapy.

### Oral Antibiotic Agent Utilization and Outcomes

There is no significant difference in oral antibiotic agents group utilization in patients who die and those who survived (Supplementary Table A1).

The distribution of antibiotic agent groups differed significantly between patients with GNB recurrence and those without recurrence (*P* < .001) (Supplementary Table A2). The recurrence rate among patients treated with oral fluoroquinolones (PO-Group 1) was significantly lower than that among patients treated with oral beta-lactams (PO-Group 2) (1.55% vs 3.20%, *P* < .001).

Further analysis of beta-lactam agents in the SDO group showed that most patients receiving oral beta-lactams were treated with cefpodoxime (49%), followed by cefadroxil (26%), amoxicillin (11%), and cephalexin (9.3%). In the overall SDO group, there was no significant difference in the distribution of antibiotic agents between patients with GNB recurrence and those without GNB recurrence (*P* = .051). However, the recurrence rate among patients treated with first-generation cephalosporins (cephalexin or cefadroxil) was higher than among those treated with a third-generation cephalosporin (cefpodoxime) (3.97% vs 2.56%, *P* = .034) (Supplementary Table A3).

### Patient Characteristics by Outcome

Recurrent GNB occurred in 14.3% (n = 14) of the 98 patients who died and in 14.1% (n = 248) of the 1756 patients who were readmitted during the 90-day follow-up period.

Survival versus death (Supplementary Table A1): patients who survived were younger (median age 72 vs 76 years, *P* = .005), more likely to be female (59% vs 39%, *P* < .001), and had lower ICU admission rates (16% vs 25.5%, *P* < .001), as well as lower CCI and LAP scores. *Escherichia coli* was the most frequently isolated pathogen among survivors (68% vs 50%), whereas *Enterobacter spp.* (2.2% vs 6.1%), *Klebsiella spp.* (13% vs 17%), *Pseudomonas spp.* (3.3% vs 7.1%), and *Serratia spp.* (1.1% vs 7.1%) were more commonly identified in non-survivors. *Escherichia coli* bacteremia was associated with lower mortality (*P* = .0035), while *Serratia spp. bacteremia* was associated with higher mortality (*P* = .0005).

Recurrent GNB versus no recurrent GNB (Supplementary Table A2): patients without recurrent GNB were more likely to be female (59% vs 46%), had lower CCI and LAP scores, and were less likely to have cancer (26% vs 33%, *P* = .012). No specific bacterial species were significantly associated with recurrent GNB.

All-cause readmission versus no readmission (Supplementary Table A4): patients who were readmitted were slightly older (73 vs 72 years, *P* < .001), less frequently female (50% vs 60%, *P* < .001), and more often admitted to the ICU (19.4% vs 15.3%, *P* < .001). They also had higher CCI and LAP scores, a greater prevalence of cancer (36% vs 24%, *P* < .001), and were less likely to have *Escherichia coli* bacteremia (59% vs 70%). Conversely, they were more likely to have bacteremia due to *Klebsiella spp.* (16% vs 12%), *Pseudomonas spp.* (5.8% vs 2.8%), and *Serratia spp.* (3.1% vs 0.9%). *Escherichia coli* bacteremia was associated with a lower risk of all-cause readmission (*P* < .001), while bacteremia due to *Klebsiella spp.* (*P* < .001), *Pseudomonas spp.* (*P* < .001), and *Serratia spp.* (*P* = .0018) was associated with increased all-cause readmission risk.

### Risk Factors

Several independent risk factors for adverse outcomes were identified in the full cohort. Male, CCI ≥3 and the present of cancer were significant independent risk factors of all-cause mortality. For all-cause readmission, male, prolonged LOS, and cancer were independently associated with increased risk. Recurrent GNB was significantly associated with male sex and CCI ≥3. (Table 4)

In the SDO group, male, CCI ≥3 and the presence of cancer were significant independent risk factors for all-cause mortality. For all-cause readmission, male, CCI ≥5, and cancer were independently associated with increased risk. Male and CCI ≥3 were the independent risk factor of recurrent GNB (Table 5).

**Table 5.** Risk Factors for Various Outcomes Within 90-Day Follow-Up: Adjusted Cox Regression Analysis for SDO Cohort

Characteristic	All-Cause Mortality		All-Cause Readmission		Recurrent GNB	
	HR (95% CI)	*P* Value	HR (95% CI)	*P* Value	HR (95% CI)	*P* Value
Index age	1.00 (.99, 1.01)	.7	1.00 (.98, 1.01)	.7	0.99 (.98, 1.00)	.2
Sex						
Female	…	…	…	…	…	…
Male	1.64 (1.06, 2.54)	.026	1.64 (1.06, 2.54)	.026	1.80 (1.35, 2.39)	<.001
Race/ethnicity						
White	…	…	…	…	…	…
Asian	1.33 (.74, 2.40)	.3	1.33 (.74, 2.40)	.3	1.36 (.93, 1.98)	.11
Black	0.67 (.24, 1.88)	.5	0.67 (.24, 1.88)	.5	1.05 (.59, 1.85)	.9
Hispanic	1.01 (.56, 1.82)	>.9	1.01 (.56, 1.82)	>.9	1.13 (.78, 1.64)	.5
Other/multiracial	1.57 (.38, 6.54)	.5	1.57 (.38, 6.54)	.5	0.80 (.25, 2.55)	.7
Charlson Comorbidity Index						
Mild (0–2)	…	…	…	…	…	…
Moderate (3–4)	2.69 (1.34, 5.41)	.006	2.69 (1.34, 5.41)	.006	1.70 (1.15, 2.53)	.009
Severe (≥ 5)	3.94 (2.11, 7.34)	<.001	3.94 (2.11, 7.34)	<.001	2.17 (1.52, 3.10)	<.001
LOS	0.98 (.79, 1.21)	.8	0.98 (.79, 1.21)	.8	1.06 (.93, 1.21)	.4
Inpatient IV antibiotic duration	1.18 (.93, 1.49)	.2	1.18 (.93, 1.49)	.2	0.99 (.86, 1.15)	>.9
ICU admission	1.21 (.70, 2.09)	.5	1.21 (.70, 2.09)	.5	1.07 (.73, 1.59)	.7
Cancer	2.91 (1.82, 4.65)	<.001	2.91 (1.82, 4.65)	<.001	1.36 (1.0, 1.86)	.054

HR, hazard ratio; LOS, length of hospital stay; SDO, step-down oral antibiotic therapy.

In the CIV group, no independent risk factors for mortality were identified. However, prolonged LOS and CCI ≥3 were significant independent risk factors of all-cause readmission, and CCI ≥5 was also associated with an increased risk of recurrent GNB (Table 6).

**Table 6.** Risk Factors for Various Outcomes Within 90-Day Follow-Up: Adjusted Cox Regression Analysis for CIV Cohort

Characteristic	All-Cause Mortality		All-Cause Readmission		Recurrent GNB	
	HR (95% CI)	*P* Value	HR (95% CI)	*P* Value	HR (95% CI)	*P* Value
Index age	1.05 (.46, 4.70)	.5	0.99 (.98, 1.00)	.010	0.99 (.97, 1.01)	.3
Sex						
Female	…	…	…	…	…	…
Male	3.80 (.99, 14.6)	.052	1.27 (1.01, 1.60)	.042	1.02 (.59, 1.78)	>.9
Race/ethnicity						
White	…	…	…	…	…	…
Asian	1.47 (.38, 5.70)	.6	1.14 (.83, 1.57)	.4	0.84 (.37, 1.92)	.7
Black	0.00 (.00, Infinity)	>.9	1.02 (.65, 1.61)	>.9	2.03 (.85, 4.86)	.11
Hispanic	0.43 (.05, 3.56)	.4	1.09 (.82, 1.47)	.5	1.26 (.64, 2.48)	.5
Other/multiracial	7.03 (.75, 66.2)	.088	1.03 (.48, 2.22)	>.9	0.00 (.00, Infinity)	>.9
Charlson Comorbidity Index						
Mild (0–2)	…	…	…	…	…	…
Moderate (3–4)	4.54 (.46, 45.1)	.2	1.49 (1.06, 2.11)	.022	1.09 (.46, 2.56)	.8
Severe (≥ 5)	6.08 (.76, 53.8)	.089	2.32 (1.73, 3.11)	<.001	2.33 (1.19, 4.56)	.013
LOS	1.08 (.84, 1.39)	.6	1.08 (1.01, 1.15)	.022	1.04 (.88, 1.24)	.6
Inpatient IV antibiotic duration	0.82 (.58, 1.16)	.3	0.95 (.88, 1.03)	.2	0.91 (.73, 1.13)	.4
ICU admission	3.40 (1.00, 11.5)	.050	1.07 (.81, 1.41)	.6	1.19 (.61, 2.32)	.6
Cancer	1.46 (.46, 4.70)	.4	1.26 (.98, 1.61)	.068	0.56 (.29, 1.11)	.10

CIV, complete course of intravenous antibiotic; HR, hazard ratio; LOS, length of hospital stay.

## DISCUSSION

Our study provides valuable real-world evidence on the management of uncomplicated GNB within an integrated healthcare system. Among 9164 patients, 90.2% received SDO antibiotic therapy, which was associated with lower rates of all-cause readmission and recurrent GNB compared to CIV therapy, with no significant difference in all-cause mortality during the 90-day follow-up period. A higher CCI score and male sex were significant risk factors for recurrent GNB. Fluoroquinolones, the most commonly used oral agents for the therapy of GNB, were associated with a lower risk of recurrent GNB, whereas beta-lactams were linked to a higher recurrent rate of GNB. These findings support the safety and effectiveness of SDO therapy for uncomplicated GNB. Strengths of this study include its large, well-defined cohort and the use of comprehensive, real-world data from an integrated healthcare system, which minimizes loss to follow-up and ensures complete outcome assessment.

Recent literature supports the use of SDO antibiotic therapy for GNB following adequate source control and clinical stability. Some studies have demonstrated that oral regimens are non-inferior to IV-only regimens and are associated with shorter hospital stays [2, 4]. Although standardized guidelines are lacking, many clinicians prefer oral antibiotic therapy for GNB due to its potential to reduce hospitalization duration, lower healthcare costs, minimize complications related to IV catheter use, and improve patient quality of life [6, 8].

The all-cause mortality rate in our cohort was 1.1%, which is lower than that reported in prior studies [9]. This difference likely reflects our exclusion of complicated GNB cases, including patients with GNB-related complications or prolonged hospitalizations or antibiotics used (≥21 days). In contrast, most previous studies did not apply such exclusions, which may account for the lower mortality observed in our cohort.

There was no significant difference in all-cause mortality between the CIV and SDO groups. However, SDO therapy was associated with lower rates of all-cause readmission and recurrent GNB within the 90-day follow-up period. Multivariate analysis identified higher CCI score and male sex as independent risk factors for recurrent GNB. Notably, the CIV group had a higher proportion of patients with elevated CCI scores and male sex, which likely contributed to their increased baseline risk of recurrent GNB.

The literature on risk factors for adverse outcomes in GNB remains limited, with considerable variation across studies [10–12]. Although some studies have proposed predictive scoring models to estimate patient outcomes [13, 14], one identified a high CCI score as a significant predictor of mortality in GNB patients [15], consistent with our findings. Most prior research has focused on short-term outcomes, such as 30-day mortality, and frequently included patients with complicated GNB. In contrast, our study evaluated risk factors for adverse outcomes over a longer 90-day follow-up period specifically in patients with uncomplicated GNB. Our analysis demonstrated that patients who developed recurrent GNB were more likely to be male, require prolonged ICU stays, and have elevated CCI and LAP scores. Similarly, patients treated with CIV therapy were more likely to be male, require ICU admission, and have higher CCI and LAP scores than those treated with SDO therapy. These findings suggest that clinicians may preferentially prescribe CIV therapy to patients perceived to be at higher risk, which may partly account for the higher rate of recurrent GNB observed in the CIV group.

Among patients readmitted to hospital during 90-day follow-up, only 14.1% experienced recurrent GNB. The majority of hospital readmissions were due to causes unrelated to recurrent GNB, suggesting that the all-cause readmission rates may be a poor indicator of antibiotic treatment failure for GNB. In our study, the 90-day all-cause readmission rate was higher in the CIV group. However, we do not interpret this as an effect of antibiotic route. The higher all-cause readmission rate is more likely attributable to the greater comorbidity burden in the CIV group, as clinicians may prefer IV therapy for sicker patients or for infections requiring IV treatment due to resistance. Consistent with this, most readmissions in our cohort were unrelated to recurrent GNB.

Our study found that the distribution of gram-negative pathogens did not differ significantly between patients with and without recurrent GNB. While certain pathogens, such as *Serratia* spp., were associated with increased all-cause mortality, and others, including *Klebsiella* spp., *Pseudomonas* spp., and *Serratia* spp., were linked to higher all-cause readmission rates, these associations likely reflect underlying patient risk factors for mortality or readmission rather than a direct causal effect of specific bacterial species in recurrent GNB.

In our study, outcomes such as all-cause mortality and recurrent GNB in the CIV group were not significantly associated with the specific categories of IV antibiotics used. However, in the SDO group, the choice of oral antibiotic influenced GNB recurrence rates. Patients treated with oral fluoroquinolones had significantly lower GNB recurrence rates compared to those receiving other oral agents, whereas oral beta-lactams were associated with higher GNB recurrence. These findings are consistent with existing literature supporting fluoroquinolones as a preferred option for bloodstream infections due to their high oral bioavailability [16–18]. In comparison, the role of oral beta-lactams in the treatment of GNB remains less well defined. Although some studies have demonstrated outcomes comparable to fluoroquinolones [3, 19–23], others, including meta-analyses, have reported higher GNB recurrence rates associated with oral beta-lactams therapy [24, 25]. Fluoroquinolones are well established as highly bioavailable oral antibiotics, whereas most oral beta-lactams have substantially lower bioavailability. One further study demonstrated that discharge on highly bioavailable agents, compared with less bioavailable agents, was associated with significantly improved clinical outcomes in patients with GNB [26].

We further explored the association between oral beta-lactam agents and recurrent GNB. In our study, nearly half of patients receiving SDO were treated with a third-generation cephalosporin (cefpodoxime), while 35.6% received a first-generation cephalosporin (cephalexin or cefadroxil). We found that the recurrence rate among patients treated with first-generation cephalosporins was higher than among those treated with a third-generation cephalosporin. Notably, the first-generation cephalosporins cephalexin and cefadroxil have high oral bioavailability, which is generally superior to that of cefpodoxime [27]. These findings suggest that differences in oral bioavailability alone cannot fully explain the higher recurrence rate of GNB observed with beta-lactams therapy. Further studies are therefore needed to better define the role of oral beta-lactams in the treatment of uncomplicated GNB.

With respect to oral TMP-SMX, prior studies suggest no significant difference in treatment failure between oral fluoroquinolones and TMP-SMX [21, 22, 24]. In our cohort, no significant association was observed between oral TMP-SMX use and GNB recurrence. However, this finding is likely limited by the small proportion of SDO patients (3.3%) who received this regimen. Additional research is warranted to further evaluate oral TMP-SMX as a potential alternative to oral fluoroquinolones for uncomplicated GNB.

This study has several limitations.

First, the median total duration of antibiotic treatment was 14 days in both CIV and SDO groups. However, growing evidence suggests that shorter antibiotic courses, such as 7 days, may be sufficient for treating uncomplicated GNB [1, 28–30]. Our study did not specifically evaluate the optimal duration of antibiotic therapy. Meanwhile, the median duration of IV antibiotic therapy in the SDO group was 4 days (range 3–5 days), which may have minimized the overall impact of SDO and agent selection on outcomes, especially if a total treatment duration of 7 days is generally sufficient as reported in other studies. This could also explain why the mortality rate in our study was substantially lower than in other studies [9]. However, mortality rates for GNB vary widely across studies [31, 32].

Second, our study did not evaluate antibiotic dosing in patients with SDO. Therefore, we are unable to determine whether treatment failure was related to insufficient oral antibiotic dosing, particularly among patients who received oral beta-lactams.

Third, as an observational and retrospective analysis, the study was inherently limited in its ability to control exposure factors, covariates, and potential confounders. Selection bias may have influenced our results. Treatment with CIV versus SDO was determined by provider decision rather than a standardized protocol. Patients in the CIV group may have lacked suitable oral options due to resistant organisms, drug allergies, or other contraindications, whereas all patients in the SDO group had appropriate oral agents available. In addition, patients with ≥21 days of hospitalization or antibiotic therapy were classified as having complicated GNB and were excluded, and some critically ill patients who would have otherwise remained in the CIV group were also excluded due to discharge to skilled nursing or hospice facilities. These factors may have contributed to residual selection bias, potentially leading to more favorable outcomes in the SDO group, such as lower all-cause readmission and recurrent GNB.

Fourth, we did not use a landmark or time-dependent analytic approach and excluded patients who died during the index hospitalization. Because patients had to survive long enough to be eligible for oral step-down therapy, this may have introduced survivor bias favoring the SDO group.

Fifth, when evaluating the association between antibiotic route (CIV vs SDO) and outcomes using Cox regression models, the low absolute number of events in our cohort, particularly the small number of all-cause mortality events, necessitated inclusion of only variables with *P* < .05 in bivariate analyses. This covariate selection approach was also specified in our research protocol. However, we acknowledge that this strategy may have excluded clinically important confounders.

Sixth, our findings were derived from the Northern California Kaiser Permanente healthcare system, where fluoroquinolones and beta-lactams were the predominant oral agents used in the SDO group. This limits the generalizability of our results to settings where different oral antibiotics are commonly used. While our study provides meaningful real-world evidence for the Northern California population, where Kaiser members account for roughly one-third of insured individuals, the applicability of these findings may be reduced in healthcare systems with different prescribing practices or resistance profiles, either nationally or internationally.

Finally, our study has additional limitations. The use of routinely collected administrative data may introduce miscoding or diagnostic errors, although systematic bias is unlikely. Furthermore, adverse effects of antibiotic therapy were not assessed due to limitations in the available data.

In conclusion, our study provides valuable insights into the management of uncomplicated GNB within the KPNC system. Step-down oral antibiotic therapy antibiotic therapy was associated with outcomes comparable to CIV therapy with respect to all-cause mortality. A high CCI score emerged as the strongest predictor of adverse outcomes, including mortality, hospital readmission, and recurrent GNB. While fluoroquinolones remain a preferred option for SDO treatment, further research is warranted to evaluate the efficacy of alternative antibiotic agents, such as beta-lactams and TMP-SMX.

## Supplementary Material

ofag200_Supplementary_Data

## Supplementary Data

Supplementary materials are available at *Open Forum Infectious Diseases* online. Consisting of data provided by the authors to benefit the reader, the posted materials are not copyedited and are the sole responsibility of the authors, so questions or comments should be addressed to the corresponding author.
