# Supplementary material for: The Use of Step-Down Oral Antibiotic Therapy for Uncomplicated Gram-Negative Bacteremia and Risk Factors for Adverse Outcomes
Source: Open Forum Infect Dis. 2026 Apr 9;13(5):ofag200. doi: 10.1093/ofid/ofag200 (PMC13175978; doi:10.1093/ofid/ofag200)
Supplement: ofag200_Supplementary_Data [file ofag200_supplementary_data.docx]

**Supplementary material**

**Supplement Table A1: Comparison of demographic and clinical characteristics between patients who died and survived within 90-day follow-up in the full cohort**

| **Characteristics** | **Death**  N = 98^[1]^ | **Survival**  N = 9,066^[1]^ | **p-value**^[2]^ | **Overall**  N = 9,164^[1]^ |
| --- | --- | --- | --- | --- |
| Index Age | 76 (66, 84) | 72 (60, 81) | 0.005 | 72 (61, 81) |
| Sex |  |  | <0.001 |  |
| Female | 38 (39%) | 5,315 (59%) |  | 5,353 (58%) |
| Male | 60 (61%) | 3,751 (41%) |  | 3,811 (42%) |
| Race/Ethnicity |  |  | 0.5 |  |
| White | 57 (58%) | 4,688 (52%) |  | 4,745 (52%) |
| Asian | 18 (18%) | 1,521 (17%) |  | 1,539 (17%) |
| Black | 4 (4.1%) | 633 (7.0%) |  | 637 (7.0%) |
| Hispanic | 16 (16%) | 2,015 (22%) |  | 2,031 (22%) |
| Other/Multiracial | 3 (3.1%) | 200 (2.2%) |  | 203 (2.2%) |
| Unknown | 0 (0%) | 9 (<0.1%) |  | 9 (<0.1%) |
| Length of hospital stay (Days) | 5.00 (4.00, 8.00) | 5.00 (4.00, 6.00) | <0.001 | 5.00 (4.00, 6.00) |
| Inpatient intravenous Antibiotic (Days) | 5.00 (4.00, 7.00) | 4.00 (3.00, 6.00) | <0.001 | 4.00 (3.00, 6.00) |
| Total Medication Duration (Days) | 15.0 (11.0, 16.0) | 14.0 (11.0, 15.0) | 0.3 | 14.0 (11.0, 15.0) |
| ICU Admission | 25 (25.5%) | 1452 (16%) | 0.018 | 1477 (16.1%) |
| Charlson Comorbidity Index |  |  | <0.001 |  |
| Mild (0-2) | 16 (16%) | 4,746 (52%) |  | 4,762 (52%) |
| Moderate (3-4) | 22 (22%) | 1,739 (19%) |  | 1,761 (19%) |
| Severe (>=5) | 60 (61%) | 2,581 (28%) |  | 2,641 (29%) |
| Laboratory Acute Physiology Score | 109 (88, 136) | 90 (67, 115) | <0.001 | 90 (67, 115) |
| Comorbidity Point Score, Version 2.5 | 79 (54, 116) | 31 (13, 60) | <0.001 | 32 (13, 61) |
| Antibiotic route |  |  | 0.2 |  |
| CIV | 13 (13%) | 888 (9.8%) |  | 901 (9.8%) |
| OSD | 85 (87%) | 8,178 (90%) |  | 8,263 (90%) |
| Pathogen of GNB |  |  | <0.001 |  |
| *Escherichia coli* | 50 (51%) | 6,147 (68%) |  | 6,197 (68%) |
| *Citrobacter spp.* | 1 (1.0%) | 117 (1.3%) |  | 118 (1.3%) |
| *Enterobacter spp.* | 6 (6.1%) | 200 (2.2%) |  | 206 (2.2%) |
| *Haemophilus spp.* | 0 (0%) | 88 (1.0%) |  | 88 (1.0%) |
| *Klebsiella spp.* | 17 (17%) | 1,163 (13%) |  | 1,180 (13%) |
| *Proteus spp.* | 1 (1.0%) | 354 (3.9%) |  | 355 (3.9%) |
| Pseudomonas | 7 (7.1%) | 302 (3.3%) |  | 309 (3.4%) |
| *Serratia spp.* | 7 (7.1%) | 98 (1.1%) |  | 105 (1.1%) |
| Multiple Infections | 1 (1.0%) | 193 (2.1%) |  | 194 (2.1%) |
| Other | 8 (8.2%) | 404 (4.5%) |  | 412 (4.5%) |
| Comorbidity of Cancer | 57 (58%) | 2,318 (26%) | <0.001 | 2,375 (26%) |
| Category of IV Antibiotic |  |  | 0.2 |  |
| IV-Group 1 | 28 (29%) | 3,569 (39%) |  | 3,597 (39%) |
| IV-Group 2 | 7 (7.1%) | 589 (6.5%) |  | 596 (6.5%) |
| IV-Group 3 | 1 (1.0%) | 64 (0.7%) |  | 65 (0.7%) |
| IV-Group 4 | 4 (4.1%) | 228 (2.5%) |  | 232 (2.5%) |
| IV-Group 5 | 0 (0%) | 15 (0.2%) |  | 15 (0.2%) |
| IV-Group 6 | 58 (59%) | 4,601 (51%) |  | 4,659 (51%) |
| Category of Oral Antibiotic |  |  | 0.3 |  |
| PO-Group 1 | 44 (45%) | 3,822 (42%) |  | 3,866 (42%) |
| PO-Group 2 | 30 (31%) | 3,505 (39%) |  | 3,535 (39%) |
| PO-Group 3 | 2 (2.0%) | 267 (2.9%) |  | 269 (2.9%) |
| PO-Group 4 | 13 (12.8%) | 890 (9.7%) |  | 903 (9.7%) |
| PO- Group 5 | 9 (9.2%) | 582 (6.4%) |  | 591 (6.4%) |

[1]: Median (IQR); N (%). [2]: Wilcoxon rank sum test; Fisher’s Exact Test with Simulated P-value

CIV: complete course of intravenous antibiotic; SDO: step-down oral antibiotic; IV: intravenous; PO: *per os* (by oral); GNB: gram-negative bacteremia

**Supplement Table A2: Comparison of demographic and clinical characteristics between patients with and without recurrent GNB within 90-day follow-up in the full cohort**

| **Characteristics** | **GNB Recurrence**  N = 248^[1]^ | **No GNB recurrence**  N = 8,916^[1]^ | **p-value**^[2]^ | **Overall**  N = 9,164^[1]^ |
| --- | --- | --- | --- | --- |
| Index Age | 72 (62, 81) | 72 (60, 81) | 0.9 | 72 (61, 81) |
| Sex |  |  | <0.001 |  |
| Female | 114 (46%) | 5,239 (59%) |  | 5,353 (58%) |
| Male | 134 (54%) | 3,677 (41%) |  | 3,811 (42%) |
| Race/Ethnicity |  |  | 0.6 |  |
| White | 121 (49%) | 4,624 (52%) |  | 4,745 (52%) |
| Asian | 47 (19%) | 1,492 (17%) |  | 1,539 (17%) |
| Black | 21 (8.5%) | 616 (6.9%) |  | 637 (7.0%) |
| Hispanic | 56 (23%) | 1,975 (22%) |  | 2,031 (22%) |
| Other/Multiracial | 3 (1.2%) | 200 (2.2%) |  | 203 (2.2%) |
| Unknown | 0 (0%) | 9 (0.1%) |  | 9 (<0.1%) |
| Length of Hospital Stay (Days) | 5.00 (4.00, 7.00) | 5.00 (4.00, 6.00) | 0.016 | 5.00 (4.00, 6.00) |
| Inpatient Intravenous Antibiotic (Days) | 5.00 (4.00, 6.00) | 4.00 (3.00, 6.00) | 0.006 | 4.00 (3.00, 6.00) |
| Total Antibiotic Duration (Days) | 14.0 (11.0, 16.0) | 14.0 (11.0, 15.0) | >0.9 | 14.0 (11.0, 15.0) |
| ICU Admission | 49 (19.6%) | 1428 (16%) | 0.12 | 1477 (16.1%) |
| Charlson Comorbidity Index |  |  | <0.001 |  |
| Mild (0-2) | 89 (36%) | 4,673 (52%) |  | 4,762 (52%) |
| Moderate (3-4) | 51 (21%) | 1,710 (19%) |  | 1,761 (19%) |
| Severe (>=5) | 108 (44%) | 2,533 (28%) |  | 2,641 (29%) |
| Laboratory Acute Physiology Score | 100 (74, 122) | 90 (67, 115) | <0.001 | 90 (67, 115) |
| Antibiotic Route |  |  | <0.001 |  |
| CIV | 52 (21%) | 849 (9.5%) |  | 901 (9.8%) |
| SDO | 196 (79%) | 8,067 (90%) |  | 8,263 (90%) |
| Pathogen of GNB |  |  | 0.081 |  |
| *Escherichia coli* | 160 (65%) | 6,037 (68%) |  | 6,197 (68%) |
| *Citrobacter spp.* | 2 (0.8%) | 116 (1.3%) |  | 118 (1.3%) |
| *Enterobacter spp.* | 10 (4.0%) | 196 (2.2%) |  | 206 (2.2%) |
| *Haemophilus spp.* | 1 (0.4%) | 87 (1.0%) |  | 88 (1.0%) |
| *Klebsiella spp.* | 38 (15%) | 1,142 (13%) |  | 1,180 (13%) |
| *Escherichia coli* | 8 (3.2%) | 347 (3.9%) |  | 355 (3.9%) |
| *Citrobacter spp.* | 10 (4.0%) | 299 (3.4%) |  | 309 (3.4%) |
| *Enterobacter spp.* | 6 (2.4%) | 99 (1.1%) |  | 105 (1.1%) |
| Multiple Infections | 8 (3.2%) | 186 (2.1%) |  | 194 (2.1%) |
| Other | 5 (2.0%) | 407 (4.6%) |  | 412 (4.5%) |
| Comorbidity of Cancer | 82 (33%) | 2,293 (26%) | 0.012 | 2,375 (26%) |
| Category of IV Antibiotic |  |  | 0.7 |  |
| IV-Group 1 | 90 (36%) | 3,507 (39%) |  | 3,597 (39%) |
| IV-Group 2 | 20 (8.1%) | 576 (6.5%) |  | 596 (6.5%) |
| IV-Group 3 | 3 (1.2%) | 62 (0.7%) |  | 65 (0.7%) |
| IV-Group 4 | 6 (2.4%) | 226 (2.5%) |  | 232 (2.5%) |
| IV-Group 5 | 0 (0%) | 15 (0.2%) |  | 15 (0.2%) |
| IV-Group 6 | 129 (52%) | 4,530 (51%) |  | 4,659 (51%) |
| Category of Oral Antibiotic |  |  | <0.001 |  |
| PO-Group 1 | 60 (24%) | 3,806 (43%) |  | 3,866 (42%) |
| PO-Group 2 | 113 (46%) | 3,422 (38%) |  | 3,535 (39%) |
| PO-Group 3 | 6 (2.4%) | 263 (2.9%) |  | 269 (2.9%) |
| PO-Group 4 | 52 (20.7%) | 851 (9.7%) |  | 903 (9.7%) |
| PO-Group 5 | 17 (6.9%) | 574 (6.4%) |  | 591 (6.4%) |

[1]: Median (IQR); N (%). [2]: Wilcoxon rank sum test; Fisher’s Exact Test with Simulated P-value

CIV: complete course of intravenous antibiotic; SDO: step-down oral antibiotic; IV: intravenous; PO: *per os* (by oral); GNB: gram-negative bacteremia

**Supplement Table A3 Step-down oral beta-lactam agents utilized among patients with and without GNB recurrence during 90-day follow-up**

| **Recurrence Status** | **GNB Recurrence**  N = 113^[1]^ | **No GNB Recurrence**  N = 3,422^[1]^ | **p-value**^[2]^ | **Overall**  N = 3,535^[1]^ |
| --- | --- | --- | --- | --- |
| Beta-lactams Agents |  |  | 0.051 |  |
| Cephalexin | 16 (14%) | 311 (9.1%) |  | 327 (9.3%) |
| Cefadroxil | 34 (30%) | 898 (26%) |  | 932 (26%) |
| Cefuroxime | 2 (1.8%) | 24 (0.7%) |  | 26 (0.7%) |
| Cefdinir | 0 (0%) | 9 (0.3%) |  | 9 (0.3%) |
| Cefixime | 0 (0%) | 3 (<0.1%) |  | 3 (<0.1%) |
| Cefpodoxime | 44 (39%) | 1,674 (49%) |  | 1,718 (49%) |
| Amoxicillin | 9 (8.0%) | 383 (11%) |  | 392 (11%) |
| Multiple | 8 (7.1%) | 120 (3.5%) |  | 128 (3.6%) |

[1]: N (%). [2] Fisher’s Exact Test with Simulated P-value

GNB: gram-negative bacteremia

**Supplement Table A4: Comparison of demographic and clinical characteristics between patients with and without all-cause readmission within 90-day follow-up in the full cohort**

| **Characteristics** | **All-cause Readmission**  N = 1,756^[1]^ | **No Readmission**  N = 7,408^[1]^ | **p-value**^[2]^ | **Overall**  N = 9,164^[1]^ |
| --- | --- | --- | --- | --- |
| Index Age | 73 (63, 82) | 72 (60, 81) | <0.001 | 72 (61, 81) |
| Sex |  |  | <0.001 |  |
| Female | 882 (50%) | 4,471 (60%) |  | 5,353 (58%) |
| Male | 874 (50%) | 2,937 (40%) |  | 3,811 (42%) |
| Race/Ethnicity |  |  | 0.006 |  |
| White | 952 (54%) | 3,793 (51%) |  | 4,745 (52%) |
| Asian | 266 (15%) | 1,273 (17%) |  | 1,539 (17%) |
| Black | 145 (8.3%) | 492 (6.6%) |  | 637 (7.0%) |
| Hispanic | 363 (21%) | 1,668 (23%) |  | 2,031 (22%) |
| Other/Multiracial | 30 (1.7%) | 173 (2.3%) |  | 203 (2.2%) |
| Unknown | 0 (0%) | 9 (0.1%) |  | 9 (<0.1%) |
| Length of Hospital stay (Days) | 5.00 (4.00, 7.00) | 5.00 (4.00, 6.00) | <0.001 | 5.00 (4.00, 6.00) |
| Inpatient Intravenous Antibiotic (Days) | 5.00 (4.00, 6.00) | 4.00 (3.00, 5.00) | <0.001 | 4.00 (3.00, 6.00) |
| Total Antibiotic Duration (Days) | 14.0 (11.0, 16.0) | 14.0 (11.0, 15.0) | 0.3 | 14.0 (11.0, 15.0) |
| ICU Admission | 340 (19.4%) | 1137 (15.3%) | <0.001 | 1,477 (16.1%) |
| Charlson Comorbidity Index |  |  | <0.001 |  |
| Mild (0-2) | 625 (36%) | 4,137 (56%) |  | 4,762 (52%) |
| Moderate (3-4) | 370 (21%) | 1,391 (19%) |  | 1,761 (19%) |
| Severe (>=5) | 761 (43%) | 1,880 (25%) |  | 2,641 (29%) |
| Laboratory Acute Physiology Score | 99 (76, 123) | 89 (65, 113) | <0.001 | 90 (67, 115) |
| Antibiotic Route |  |  | <0.001 |  |
| CIV | 298 (17%) | 603 (8.1%) |  | 901 (9.8%) |
| OSD | 1,458 (83%) | 6,805 (92%) |  | 8,263 (90%) |
| Pathogen of GNB |  |  | <0.001 |  |
| *Escherichia coli* | 1,028 (59%) | 5,169 (70%) |  | 6,197 (68%) |
| *Citrobacter spp.* | 27 (1.5%) | 91 (1.2%) |  | 118 (1.3%) |
| *Enterobacter spp.* | 53 (3.0%) | 153 (2.1%) |  | 206 (2.2%) |
| *Haemophilus spp.* | 16 (0.9%) | 72 (1.0%) |  | 88 (1.0%) |
| *Klebsiella spp.* | 285 (16%) | 895 (12%) |  | 1,180 (13%) |
| *Proteus spp.* | 67 (3.8%) | 288 (3.9%) |  | 355 (3.9%) |
| *Pseudomonas spp.* | 101 (5.8%) | 208 (2.8%) |  | 309 (3.4%) |
| *Serratia spp.* | 36 (2.1%) | 69 (0.9%) |  | 105 (1.1%) |
| Multiple Infections | 45 (2.6%) | 149 (2.0%) |  | 194 (2.1%) |
| Other | 98 (5.6%) | 314 (4.2%) |  | 412 (4.5%) |
| Comorbidity of Cancer | 627 (36%) | 1,748 (24%) | <0.001 | 2,375 (26%) |
| Category of IV Antibiotic |  |  | <0.001 |  |
| IV-Group 1 | 533 (30%) | 3,064 (41%) |  | 3,597 (39%) |
| IV-Group 2 | 162 (9.2%) | 434 (5.9%) |  | 596 (6.5%) |
| IV-Group 3 | 15 (0.9%) | 50 (0.7%) |  | 65 (0.7%) |
| IV-Group 4 | 47 (2.7%) | 185 (2.5%) |  | 232 (2.5%) |
| IV-Group 5 | 3 (0.2%) | 12 (0.2%) |  | 15 (0.2%) |
| IV-Group 6 | 996 (57%) | 3,663 (49%) |  | 4,659 (51%) |
| Category of Oral Antibiotic |  |  | <0.001 |  |
| PO-Group 1 | 648 (37%) | 3,218 (43%) |  | 3,866 (42%) |
| PO-Group 2 | 655 (37%) | 2,880 (39%) |  | 3,535 (39%) |
| PO-Group 3 | 49 (2.8%) | 220 (3.0%) |  | 269 (2.9%) |
| PO-Group 4 | 298 (17.2%) | 605 (8.5%) |  | 903 (9.7%) |
| PO-Group 5 | 106 (6.0%) | 485 (6.5%) |  | 591 (6.4%) |

[1]: Median (IQR); N (%). [2]: Wilcoxon rank sum test; Fisher’s Exact Test with Simulated P-value

CIV: complete course of intravenous antibiotic; SDO: step-down oral antibiotic; IV: intravenous; PO: *per os* (by oral); GNB: gram-negative bacteremia
